# A Soft Five-Fingered Hand Actuated by Shape Memory Alloy Wires: Design, Manufacturing, and Evaluation

**DOI:** 10.3389/frobt.2020.608841

**Published:** 2020-12-07

**Authors:** Filomena Simone, Gianluca Rizzello, Stefan Seelecke, Paul Motzki

**Affiliations:** Systems Engineering, Faculty of Natural Sciences and Technology, Saarland University, Saarland, Germany

**Keywords:** robotic hand, SMA wires, soft hand, metal muscles, shape memory alloys

## Abstract

This work presents a novel five-fingered soft hand prototype actuated by Shape Memory Alloy (SMA) wires. The use of thin (100 μm diameter) SMA wire actuators, in conjunction with an entirely 3D printed hand skeleton, guarantees an overall lightweight and flexible structure capable of silent motion. To enable high forces with sufficiently high actuation speed at each fingertip, bundles of welded actuated SMA wires are used. In order to increase the compliance of each finger, flexible joints from superelastic SMA wires are inserted between each phalanx. The resulting system is a versatile hand prototype having intrinsically elastic fingers, which is capable to grasp several types of objects with a considerable force. The paper starts with the description of the finger hand design, along with practical considerations for the optimal placement of the superelastic SMA in the soft joint. The maximum achievable displacement of each finger phalanx is measured together with the phalanxes dynamic responsiveness at different power stimuli. Several force measurement are also realized at each finger phalanx. The versatility of the prototype is finally demonstrated by presenting several possible hand configurations while handling objects with different sizes and shapes.

## Introduction

The design of a device able to reproduce all the functionalities of a human hand has been a challenge for many researchers since the late 1950'. Up to date, the most performant prototypes (in terms of force, speed, and versatility) are designed with a rigid structure, and driven by electric motors. Remarkable examples are represented by the “iLimb” (Connolly, 2018), designed by Touch Bionics in the 2009, the “Bebionic” (Medynski and Rattray, 2001) invented by RSL Stepper in the 2011, the “Michelangelo” hand (Miguelez, 2011) produced by Otto Bock in 2012, and many others (Kawasaki et al., 2002; Lotti et al., 2005; Cipriani et al., 2011; Shadow Robot Company Shadow., 2013; Jones and Stopforth, 2016). Despite the high performances achieved by these devices, they have encountered high rejection rates by users mainly due to the rigidity of the artificial hands, their heavy weight, and their noisy motion (Kyberd et al., 2004; Dollar and Howe, 2006; Biddiss et al., 2007; Trivedi et al., 2008).

Soft robotic hands represent a valuable alternative to this technology (Dollar and Howe, 2006). The increased compliance of these devices confers an intrinsic robustness to manipulation and, at the same time, safe features which are highly desirable in human collaborative environments (Cianchetti and Laschi, 2016). A first example of flexible and underactuated system is presented in Dollar and Howe (2006). In here, the authors introduce a two-finger gripper with reconfigurable joints, actuated by two DC motors. In Deimel and Brock (2013), a three-fingered gripper made of silicone layers is presented. The structure of this prototype has no joints. The motion is directly related to the silicone deformation, induced by compressed air used for actuation. Inexpensive multi-fingered hands are introduced in (Deimel and Brock, 2016; Homberg et al., 2019). The structure of these prototypes is realized thought an injection molding process, via a systematic and fast procedure. The result is a hollow rubber finger, which can be deformed with compressed air.

From the above discussion, it can be seen how most of the current soft hand prototypes are driven through pneumatic actuators. Even if pneumatic technology represents the most used and effective solution in soft robotics, it exhibits several limitations when used for hand prosthesis applications. The requirement of a compressed air source, together with its transportation systems (e.g., tubes, valves), inevitably increases the weight of the overall prototype, thus it is unsuitable for portable devices. Recently, a number of researchers started using electric motors in combination with deformable finger joints to design soft prostheses. The biggest advantage of this technology, compared to pneumatics, is represented by its compactness. One relevant example is exposed in Hussain et al. (2018), in which each finger joint of a five-fingered hand is realized with a flexible thermoplastic polyurethane. The actuation is provided by a DC motor, which transmits its motion to each joint in the hand through tendons. In Catalano et al. (2014), a 19-joint hand has been developed using elastic bands for connecting each finger phalanx, in order to enable soft features in the device. We point out how, despite the high compliance and performances reached by those hand, their heavy weight and their noisy motion prevent them to be fully appreciated by amputee (Trivedi et al., 2008).

Shape memory alloys (SMA) represent an alternative actuation technology which can overcome several limitations of the previously described solutions, in terms of noise, weight, and structure simplicity. These active transducers, typically consisting of Ni–Ti alloys, undergo a phase transformation when exposed to heat (Lagoudas, 2008). Macroscopically, this phase transformation results into a contraction (on the order of 4–8%). When used in engineering applications, SMAs are typically shaped as thin wires. In this way, the heating can be effectively produced by sending an electric current through the wire. At the same time, the wire geometry can be easily integrate in small spaces. Furthermore, if the electrical resistance of the SMA wire is measured during actuation, it can be used to reconstruct the wire length without using any additional sensor in the device (Furst and Seelecke, 2012). This simultaneous actuation and sensing feature, often named “self-sensing,” makes SMA wires comparable to human nerves which are capable of feeling and transmitting information at the same time.

Over the past years, several rigid hand structures driven by SMA wires have been developed (Bundhoo et al., 2009; Lee et al., 2012; Andrianesis and Tze, 2015; Simone et al., 2017). However, so far, none of them can be considered advanced enough to support amputees in typical real life operations, mainly due to their limited performance in terms of dexterity and speed (Kyberd et al., 2004; Lee et al., 2012). More recently, SMA technology has also been employed for designing soft robots as well as soft hand prototypes (Verl et al., 2015; Rodrigue et al., 2017). One of the few relevant example is given by Wang and Ahn (2017), where a hand prototype made of PDMS is presented. In here, deformable shape memory polymer joints are actuated by SMA wires, casted in the PDMS itself.

On the one hand, by analyzing the recent trends in literature, it can be noted that soft robotic hands represent a big improvement toward the development of a human-like hand device. On the other hand, very few soft hand prototypes driven by SMA wires have been developed so far. We point out how, in contrast to the prototypes based on stiff joints, current deformable hands lack the gripping force needed to accomplish daily life operations (Rodrigue et al., 2017). In order to overcome this issue, the present work introduces a new soft hand prototype actuated by SMA wires. A previous version of the three-fingered SMA actuated hand was presented in Simone et al. (2017). Despite showing remarkable performance, the hand developed in Simone et al. (2017) suffers from a number of limitations, mainly in terms of force, since the maximum achievable force is in the range of 1.5 N per finger, and versatility, mostly due to its structure design. The novel hand prototype developed in this work permits to achieve higher versatility and higher forces with respect to the state-of-the-art prototypes (Lee et al., 2019), while ensuring at the same time a soft structure, a limited weight, and silent actuation. This result is achieved by using bundles of SMA wires (100 μm diameter) as driving element for each finger phalanx. In order to obtain a lightweight and robust system, each wire is welded to a very small metal sheet, and then placed inside the structure along the phalanxes (Scholtes et al., 2018). This solution permits to directly apply the wire force at the finger phalanx by avoiding the use of any transmission mechanism, thus mimicking the muscle fibers/tendons inside the human hand. The use of bundles composed of many wires permits to achieve forces comparable to a human hand. In addition, by arranging the bundles in a protagonist–antagonist configuration and using optimized pulsed control scheme, higher actuation speed can be achieved in relation to standard SMA-spring mechanisms (Fu et al., 2009). The described solution enables full opening-closing motion and a high versatility. In order to increase the overall structure compliance, flexible joints made of superelastic SMA wires are designed. This frictionless solid-state solution enables soft features in the prototype. An easy-to-assemble structure, which does not require any complex handcraft work, is proposed and designed. In this way, the entire prototype manufacturing can be potentially integrated in a production chain. In order to highlight the prototype performances, several experiments are performed to evaluate the finger force, motion range, velocity, and grasping capabilities. We point out that an early version of this research has been presented in a conference paper (Simone et al., 2018). More specifically, in Simone et al. (2018) an early SMA finger prototype activated by SMA is presented. The SMA bundles are realized through a winding process, generally used for manufacturing coils. Due to manufacturing problems and to the contact between each SMA wire in the bundles, the demonstrated motion of the prototype is extremely different from the human one (Tubiana et al., 1998). This paper aims at improving and extending the work introduced in Simone et al. (2018), by presenting:

- a more compact finger structure;
- an improved flexible joint configuration, arranged according to a X-shape;
- the full hand structure;
- a new and systematic method to manufacture the SMA bundle (welding process);
- several force measurements;
- systematic measurements of the motion range of each finger phalanx;
- a pulse method for high-speed activation of the SMA fingers;
- several hand grasping configurations while handling different size and shape object.

The remainder of the paper is organized as follows. Section Design and Models describes the design of the full hand and its fingers. Section Fabrication provides information about the prototype fabrication procedure. In section Results and Discussion, several experiments are performed with the goal of evaluating the prototype performances in terms of force, motion, reactiveness, and grasping capabilities. Finally, in section Conclusions, some concluding remarks and future research directions are outlined.

## Design and Models

**Figure 1:**
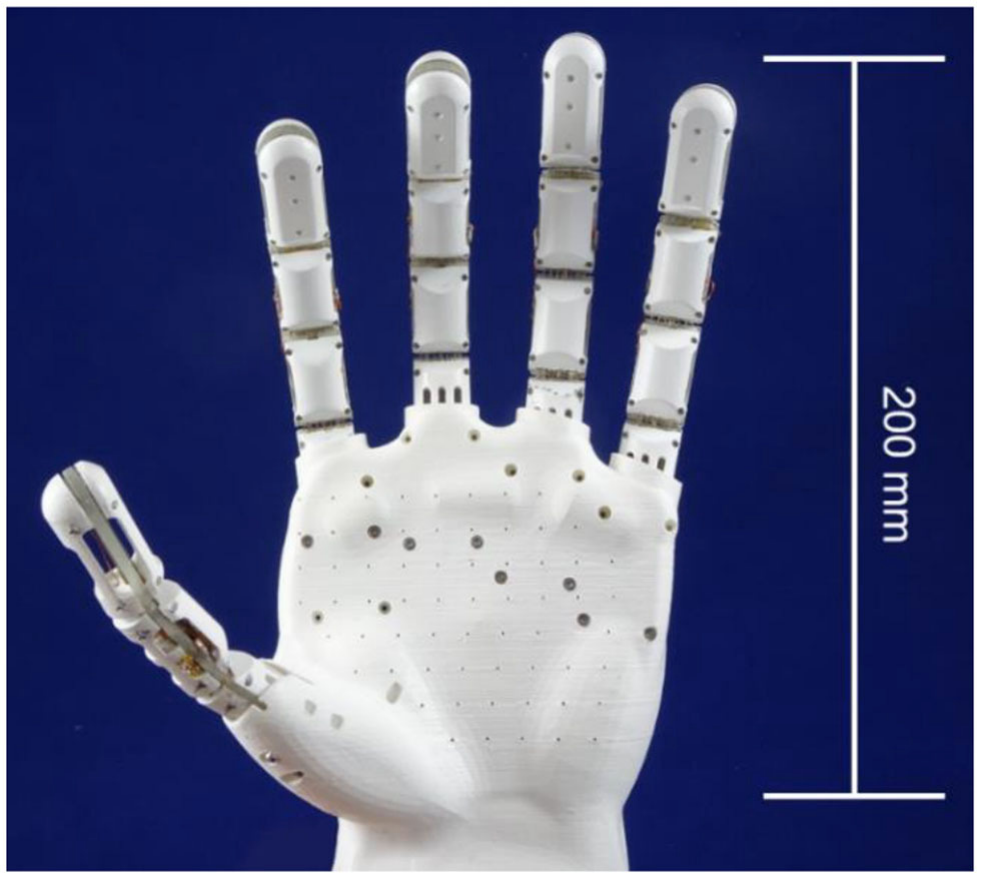
Picture of the five-fingered SMA hand.

The hand is the most complex and articulated part of the human body. It is composed of a 27 bones and 17 articulations, resulting in an overall number of 23 degrees of freedom (DOF) (Tubiana et al., 1998). In order to design a bio-inspired prosthesis which is also functional and simple, the DOFs not strictly related to a grasping motion are neglected in this work (Tubiana et al., 1998). The result is a modular 14 DOF prototype, depicted in Figure 1. It consists of a palm and five fingers, connected to the entire structure via screws. In order to simplify the design and ensure at the same time an effective grasping, the thumb is designed with an angle of 90° with respect to the hand palm surface. The system CAD is first realized in SolidWorks, and then 3D printed. In particular, the fingers are 3D printed with an Object Connex, using Object Vero White Plus Full Cure 835 as printing material, while the palm is 3D printed with an Ultimaker 3 Extended using white PLA as printing material. The structure design is dimensioned with respect to the average size of an adult human hand, as reported in Table 1, and shaped according to the human hand morphology. To ensure a lightweight structure, the hand and the fingers are left internally hollow.

**Table 1:** SMA hand system dimensions.

**Coefficient**	**Value (mm)**
**Middle/Index/Ring/Little finger**	
Finger top phalanx length	37
Finger middle phalanx length	29
Finger bottom phalanx length	30
Finger stand length	104
Finger width	16
Finger thickness	15
**Thumb finger**	
Finger top phalanx length	37
Finger bottom phalanx length	29
Finger stand length	90
Finger width	16
Finger thickness	15
**Hand**	
Hand full length	200
Hand palm width	116
Hand wrist width	85
Hand palm length	110
Hand wrist length	60
**SMA bundle**	
Tip prot. Bundle length	55
Middle prot. Bundle length	165
Bottom prot. Bundle length	116
Tip ant. Bundle length	61.5
Middle ant. Bundle length	170
Bottom ant. bundle length	122

### Finger Design

**Figure 2:**
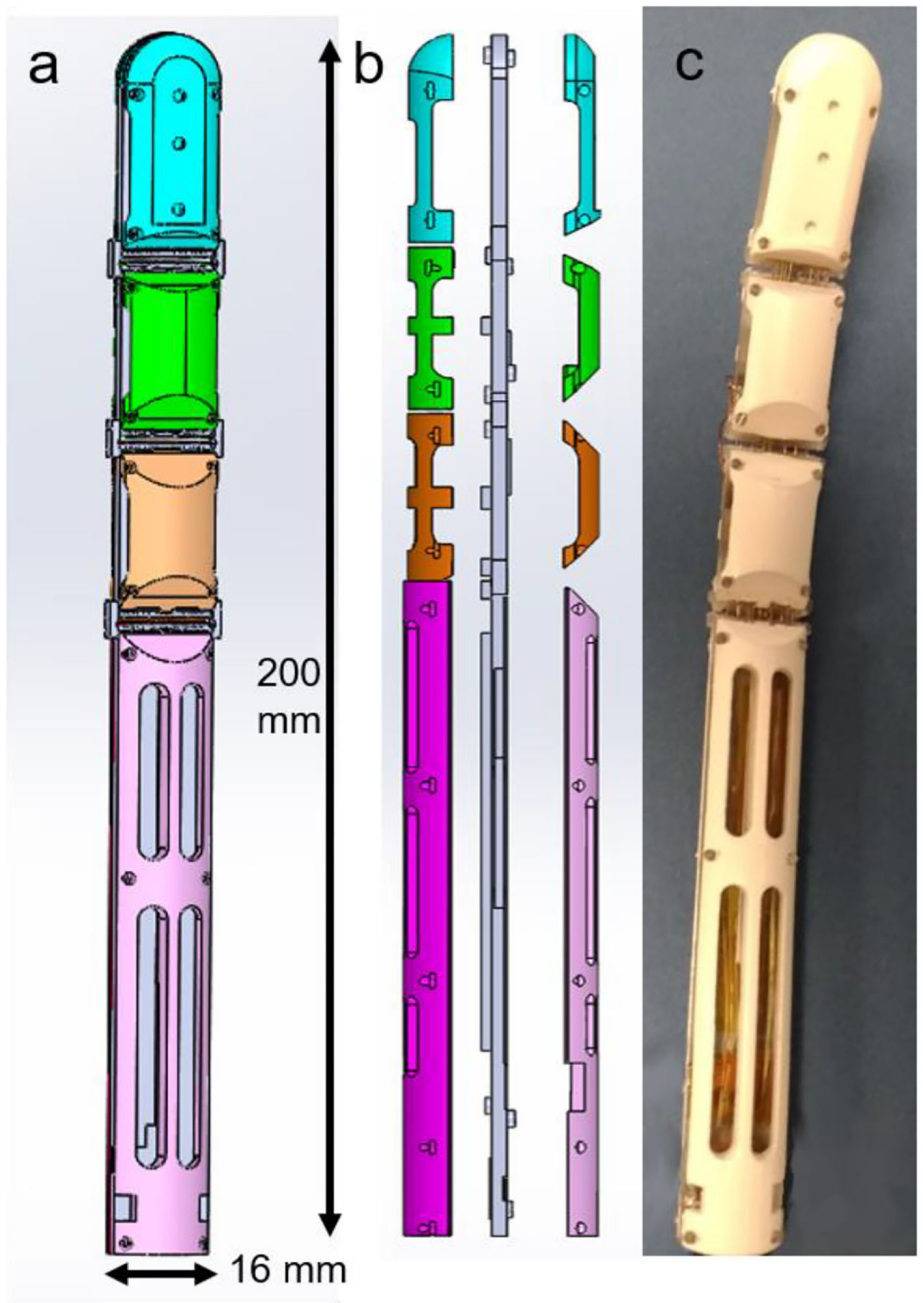
Finger structure. **(a)** CAD model of the full finger structure. Each phalanx is depicted with a different color. **(b)** Finger side split view. In gray the central part, where the SMA wires are fixed. The colored parts represent the external cover, which protects the SMA wires. **(c)** Printed and assembled finger prototype.

Each finger has a bio-inspired and modular structure. It is formed by three phalanxes and a stand, which represent the finger bone inside the palm, as shown in Figure 2.

Phalanxes with a hollow structure are designed, in order to increase the overall lightness. At the same time, to avoid undesired deformations, an arc shape pattern is used to design the finger cavities. Each finger has a modular structure composed of nine parts, i.e., the external ones (colored parts in Figure 2b), which have mostly a protection function, and the central parts (depicted in gray in Figure 2b), which encapsulate all the SMA wires. In addition, the external parts also allow to constrain the finger bending and stretching motion. Biologically, the human fingers are actuated by muscles located inside the arm and connected to each phalanx through tendons. Taking inspiration from the human biology, the SMA wires are collocated along the finger structure. When a SMA is actuated via an electric current, it contracts similarly to an artificial muscle or tendon, and a motion is induced in the finger phalanx. During the contraction, each SMA wire changes its electrical resistance according to the current length. Therefore, it is possible to relate its change in resistance to the finger motion and rotation (Simone et al., 2017). According to this behavior, the SMA wires act also as nerves.

Several SMA wire diameters are available on the market, ranging from 25 μm up to 500 μm (Fumagalli et al., 2009). In general, a thick wire is able to exert high forces, but requires also a long cooling time. Thanks to a better surface per volume ratio, thinner wires require a shorter cooling time than thicker ones, but are not able to exert high forces. For these reasons, in order to achieve forces values in the same range of human ones (Tubiana et al., 1998), bundles of 100 μm diameter SMA wires are used in this work (Fumagalli et al., 2009). Each SMA bundle is composed of nine wires welded to a 10 mm × 5 mm × 1 mm stainless steel sheet, according to the procedure described in Scholtes et al. (2018).

**Figure 3:**
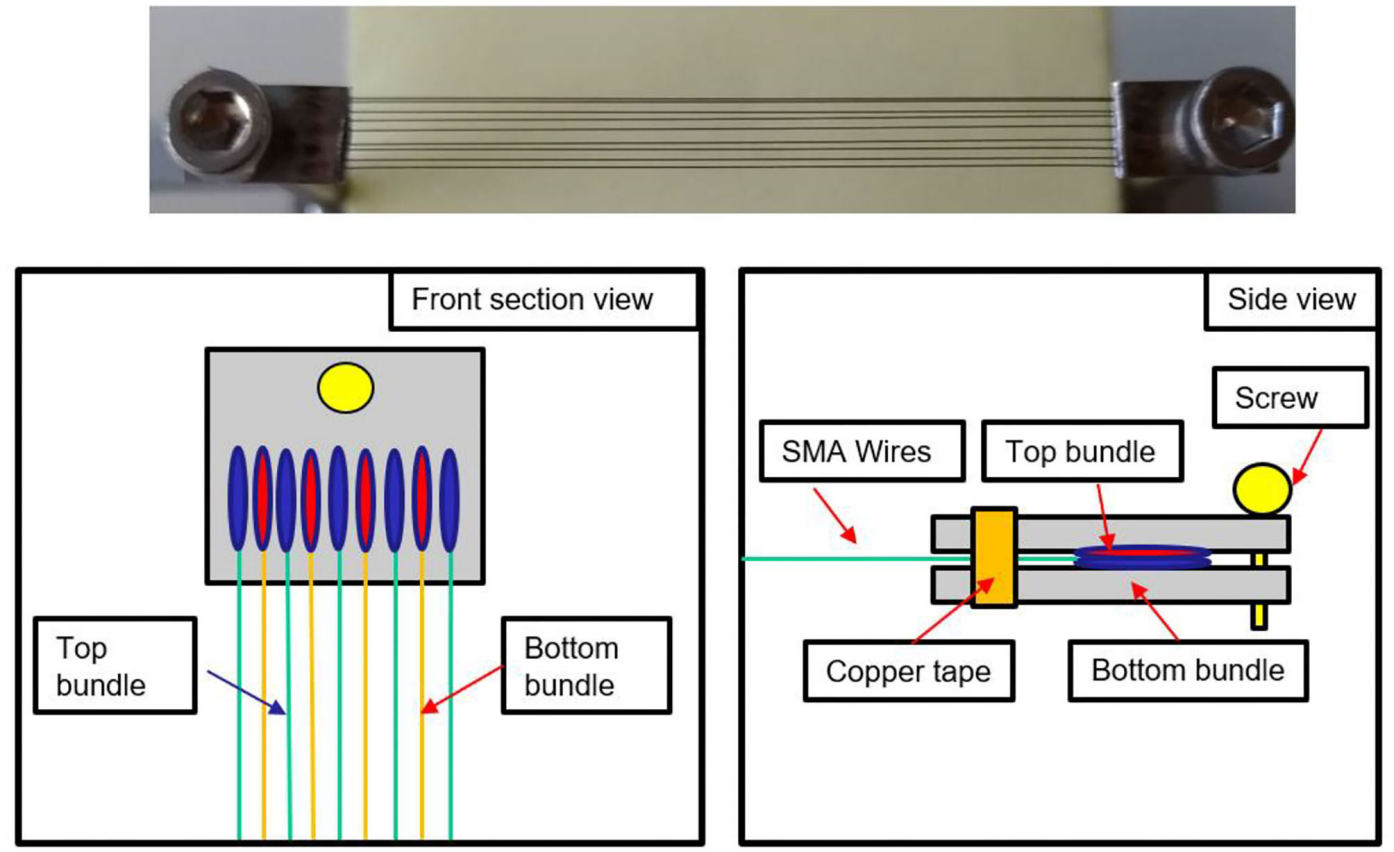
Design of the “sandwich” bundle. (Top view) Figure of the welded “sandwich bundle”. (Bottom view) Sketch of the bundle structure. All its parts are highlighted.

This process permits to systematically produce bundles of SMA wires having a repeatable and reliable behavior, since all transducer are in the same state during welding. Due to technical limitations related to the welding process, each SMA wires needs to have a minimum distance of 1 mm with its two neighbor wires. Therefore, only five SMA wires can be welded on a 5 mm wide metal sheet. In order to maximize the number of wires, a “sandwich” shape bundle is manufactured, formed by two metal sheets laying on each other, having four and five wires, respectively (as shown in Figure 3). According to Figure 3, the wires in the top bundle are properly placed in order not to overlap with any wire of the bottom bundle. A copper tape is then wrapped around the two metal sheets at both sandwich bundle ends, in order to hold the sub-bundles together and power them at the same time. Metal screws are used to fix it to the finger structure. In order to enable the motion, a protagonist-antagonist configuration is adopted. This solution permits to achieve faster actuation speed in comparison to a conventional SMA-spring configuration, ensuring a stretching (carried out by antagonist wires) and bending (by protagonist wires) motion of each phalanx depending on which bundle is activated (Andrianesis and Tze, 2015). Each finger is designed in order to achieve the desired motion range by exploiting a SMA strain of only 3.5%, which represents the safe value for high lifetime (Fumagalli et al., 2009). The SMA wires dimensioning is obtained by means of kinematic and dynamic models formulated in Simone et al. (2017, 2019).

### Soft Joints Design

In order to enable a soft behavior in the hand prototype, each finger joint is designed to be flexible. As main requirement, the joint has to be designed to fit in the center part of the SMA finger (Figure 2b), which is 2.5 mm thick. From the literature, we know that several elements such as steel trusses (Bunget and Seelecke, 2007) or polymer strips (Catalano et al., 2014) can be integrated in a structure and act as soft joint.

Despite their effectiveness, these solutions are hard to manufacture in an accurate way (i.e., steel trusses) (Bunget and Seelecke, 2007). In addition, it is difficult to achieve their miniaturization while ensuring effective compensation of all undesired moments, especially when considerably high forces act on the structure (i.e., polymer strips). In order to design a flexible joint which is durable, easy to manufacture, easy to integrate in small spaces, and at the same time, able to sustain considerable forces, superelastic SMA wires are chosen as passive components to connect each finger phalanx. These Ni-Ti alloys are characterized by a high lifetime, and are commercially available already shaped as wire (Fumagalli et al., 2009). On the one hand, conventional actuating SMA wires are manufactured in such a way their austenite finish temperature is on the order of 90°C. In this way, the transformation from martensite to austenite, and the subsequent length contraction, can be effectively triggered by an electric current. On the other hand, superelastic SMA wires are in full austenitic state already at room temperature. When subject to a mechanical stress, they undergo a transformation from austenite to martensite which is completely reverted as soon as the load is removed. By means of this effect, they can sustain an elastic deformation up to 10% (Lagoudas, 2008). This feature makes superelastic SMAs suitable to be integrated as flexible joints in soft robotics structures. In order to preserve the optimized hand design as in Simone et al. (2019), the hinge joints are directly replaced by the superelastic SMA wires located along the joint vertical axis.

In this configuration, the superelastic wires exert a passive stretching force in the structure, thus working against the protagonist SMA bundles. This design solution permits to decrease the number of active antagonist SMA bundles, decreasing also the amount of input power needed to actuate the fingers.

**Figure 4:**
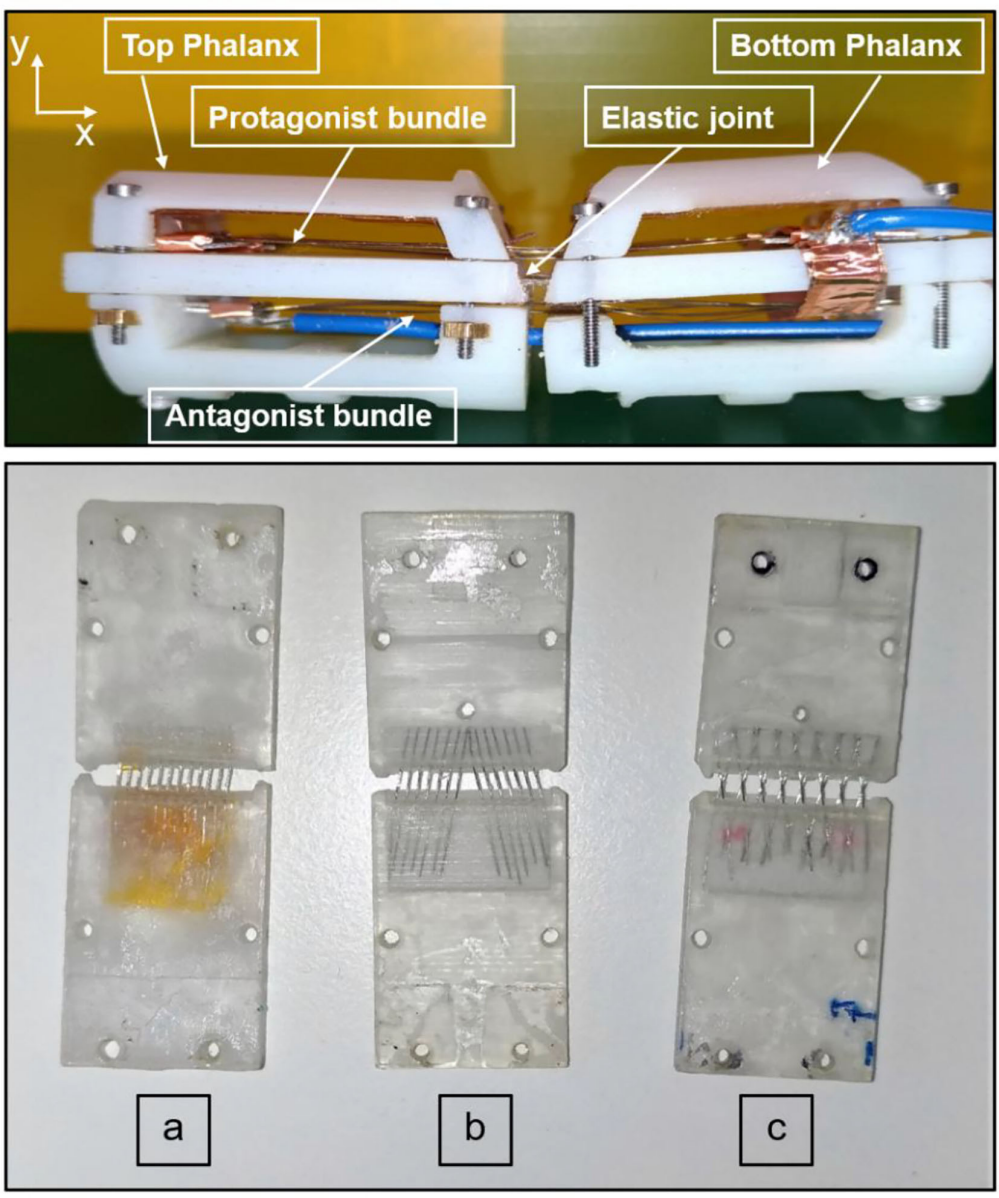
(Upper part) Two-phalanx finger prototype. (Lower part) Superelastic SMA wires arrangement on a single phalanx. **(a)** vertical arrangement. **(b)** inclined arrangement. **(c)** x-shape arrangement.

In order to design a flexible joint which is also stiff enough to sustain the structure and provide a restoring force to the protagonist wires, different superelastic SMA wire topologies are tested. To validate the different concepts, the two-phalanx finger prototype shown in Figure 4 (upper part) is used. This prototype is composed of two central parts, in which three actuating bundles are located (two protagonist and one antagonist, respectively).

Four external parts are also used to protect the internal structure and to guide the actuated SMA wires bundles (blue and green parts in Figure 2b). The central parts are connected by a soft joint, while the external ones are held together through screws. This prototype represents the tip and middle phalanxes of the finger, shown in Figure 2b (in blue and green colors). As a first solution, the superelastic SMA wires are vertically arranged, each wire parallel to the others. After a first test, it has been noticed that this topology does not permit to compensate all undesired deformations induced in the soft joint by the actuated SMA wires. When activating the protagonist SMA bundles, after an initial rotation around the *z*-axis (see Figure 4, upper part), an additional rotation around the *x*–*z* axes is induced to the top phalanx. This is due to unavoidable asymmetries in the system, as a result of inaccuracies of the 3D printing process, as well as to the high stress state generated by the actuated bundles, which makes the wires in the joint collapse into a new preferred configuration. This effect can be easily justified by comparing each superelastic SMA wire to a beam. When the SMA bundles are actuated, a force acting in the *x*-direction (i.e., the beam main axis) is applied. According to classical mechanics, a bending motion is generated at first. As soon as the overall load overcomes the critical load, each beam collapses in a buckling mode. This leads not only to an undesired top phalanx motion, but also to a permanent deformation of the superelastic wires (slightly visible in Figure 4a, lower part), which makes the SMAs in the joint tilt on one side.

In order to prevent this undesired phenomenon, an inclined arrangement is considered as a second possible design solution (Figure 4b). Preliminary experiments demonstrate that this configuration permits to effectively compensate lateral moments (details are omitted for conciseness). On the other hand, the top phalanx is still unable to perform a perfect rotary motion. This is due to the fact that, when actuated, the protagonist SMA wires induce high stresses that favor a reconfiguration of the soft joint. This leads the superelastic SMA wires to collapse on themselves, since a buckling in the *x*–*y* direction is induced.

In order to avoid this phenomenon, a X-shaped topology is finally considered (Figure 4c, lower part).

Several tests demonstrate that this design is able to compensate every major undesired moment, while still allowing a slight lateral tilt if a force is acting on the phalanx side. When the protagonist SMA bundles are activated, a rotation is induced around the middle point of every beam. In order to find the ideal number and size of superelastic SMA wires to be inserted into the joint, several prototypes are designed and tested. The use of thick wires increases the joint stiffness and, therefore, the achievable bending angle becomes smaller. Moreover, the mounting process becomes harder since a high rigidity of the wires contrasts the overlapping process needed to manufacture the X-shape joint. On the other hand, the use of thin SMA wires prevents the compensation of the stress induced during the activation of the protagonist bundles, leading to a reduced lifetime and to several damages at the wires overlapping points.

By accounting for all these factors, and after comparing different wire diameters among the ones available on the market (25–500 μm) (Fumagalli et al., 2009), superelastic SMAs having a 200 μm diameter are chosen for the final design.

### Fabrication

**Figure 5:**
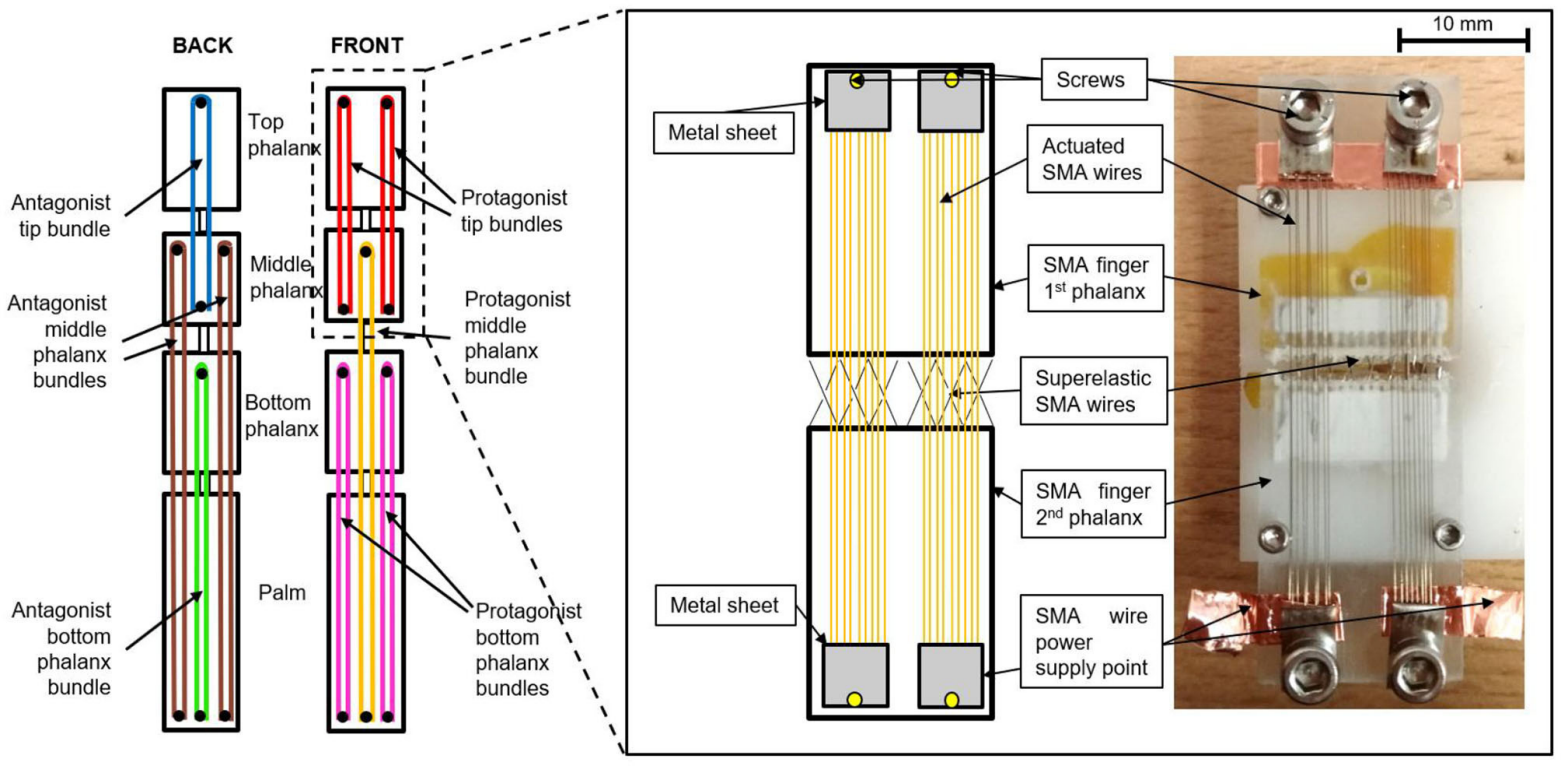
(Left-hand side) SMA bundles arrangement inside the finger structure. (Right-hand side) Comparison between design and prototype. The electrical connections are also displayed.

The fabrication of the SMA actuated hand consists of several steps, listed in the following:

- Step1—the finger structure is first designed in Solidworks, and then 3D printed with an Object Connex 500;
- Step 2—the printed structure is cleaned with isopropanol. Superelastic SMA wires having a 200 μm diameter are inserted in the central part, along small holes designed at both end of each phalanx, following the pattern displayed in Figure 5, right-hand side. High temperature epoxy glue is then used to fix them in the structure;
- Step 3—two bundles are manufactured according to the welding process described in Scholtes et al. (2018), having five and four SMA wires with a 100 μm diameter, respectively. The bundles are then overlapped as shown in Figure 3, in order to have the wires laying on the same plane. A copper tape, having conducting glue on one side, is then used to hold the two bundles in the desired position. The procedure is repeated for all nine bundles (Figure 5, left-hand side). The length of each bundle is reported in Table 1.
- Step 4—antagonist and protagonist bundles are mounted in the finger structure, and copper tape is then used to realize electrical connections (Figure 5, right-hand side). According to the chosen SMA arrangement, all the wires in a “sandwich bundle” are connected in parallel both electrically and mechanically. When two “sandwich bundles” are used to activate the same phalanx, they are designed to be electrically in series and mechanically in parallel, e.g., the bundles in red, magenta, and brown shown in Figure 5, left-hand side. This choice enables to achieve higher forces at the phalanx, while fully exploiting the available space inside the structure.

It is worthwhile noting that the tip bundle is not connected to the palm. This arrangement permits to save space in the structure, thus making it available for the bottom SMA bundle. The presented bundle arrangement provides full control of each phalanx, since the activation of each single bundle leads to the motion (stretching or bending) of one different phalanx.

At the same time, this solution ensures a shorter wire length for each bundle. For a comparison, a single 300 mm long protagonist SMA wire and a single 500 mm long antagonist one would be required to allow full bending and stretching of the structure, respectively. With this design, the prototype size would have been unsuitable for prosthetic applications. Therefore, the bundle topology has been designed in order to achieve a trade-off between space constraints, desired motion range, and exerted force. The external structural pieces are mounted around the finger central parts, in order to protect the wires and the connections. In general, hand prostheses presented in literature have a quite complex structure, and their manual assembly often requires a significant effort (Belter et al., 2013). On the other hand, the procedure to assemble the SMA Soft Hand, described above, involves simple and systematic steps that do not require any complex manual work. Indeed, the realization of the SMA bundle has been already full automatized (Scholtes et al., 2018), and the assembly of the entire structure is quite straightforward. For this reason, the fabrication of the entire hand prototype could be easily integrated in an automatic production chain, avoiding any efforts from a human worker.

## Results and Discussion

The evaluation of the actuation performance of a single finger is first presented in this section, in terms of force, bending angle, and actuation speed. Subsequently, the grasping capabilities of the complete hand actuators are illustrated.

### Finger Force

**Figure 6:**
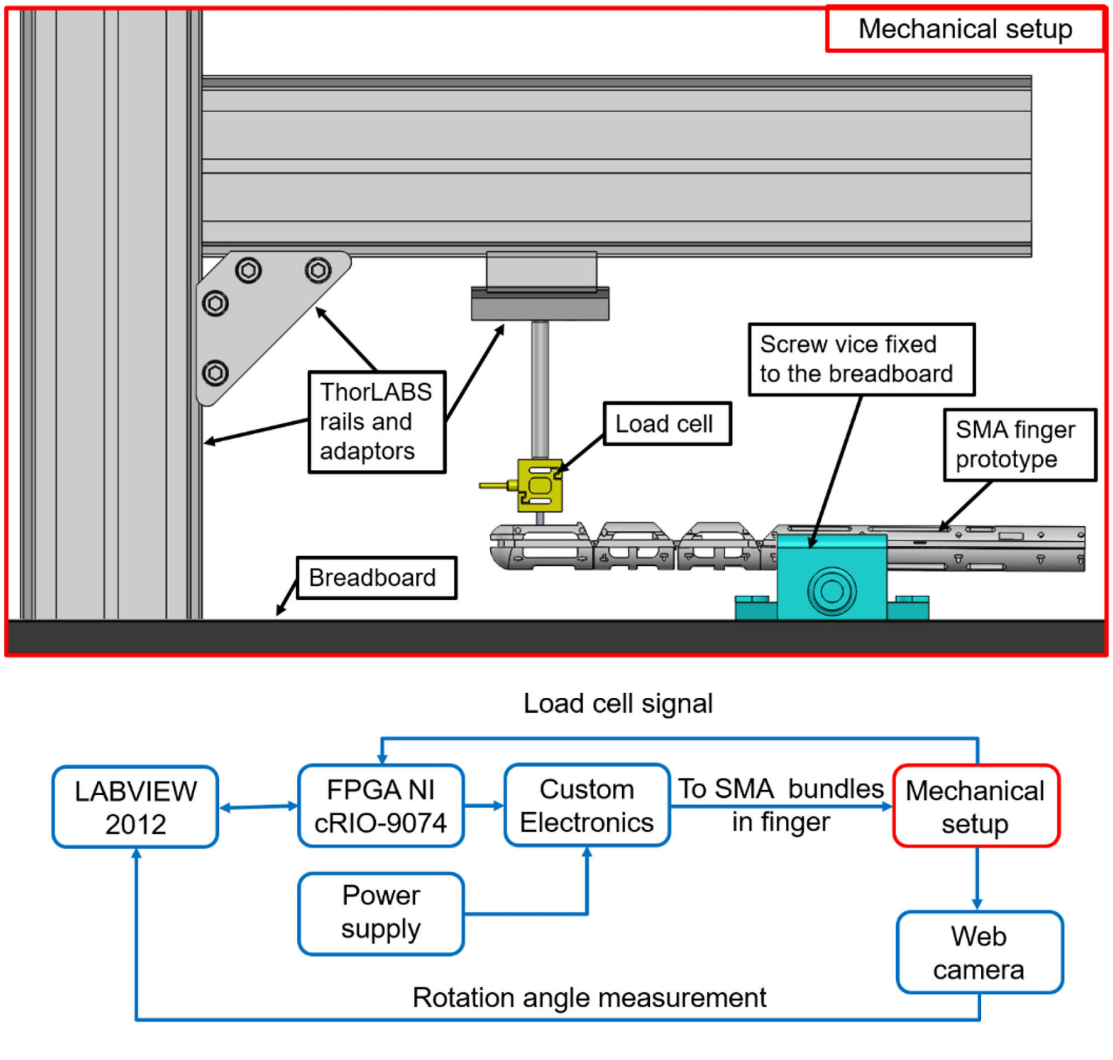
(Lower part) Force measurement diagram. (Upper part) Detail on the mechanical setup.

In this section, force measurements are realized in order to evaluate the prototype capabilities. Since all of the hand fingers have the same structure and wire arrangement, only the force of a single finger is evaluated. The experimental test bench is shown in Figure 6. It consists of the SMA finger, a vice screw, a Futek LSB 200 load cell, and ThorLABS adapters. The vice screw is anchored to a black breadboard through screws, and holds the finger prototype in correspondence of the palm part (Figure 5, left-hand side).

The load cell is connected to the ThorLABS adapters, in order to easily adjust its position in relation to the desired force evaluation point. Both load cell and finger prototype are connected to a FPGA National Instruments cRIO-9074 real-time data acquisition system, interfaced with LabVIEW 2012. The FPGA is connected with a NI 9472, a switch module able to supply up to 30V DC, and a NI 9237, which represent the hardware interface between any load cell and LABVIEW. A custom electronic board is developed in order to drive each bundle independently. By connecting each MOSFET in the board with an output of the NI 9472 module, it is possible to drive the corresponding SMA bundle independently of the other ones. In all the experiments, the electric power is chosen as control input for the SMA wires. In fact, differently from voltage and current, the electric power affects the SMA temperature in a proportional way (Furst and Seelecke, 2012). As mentioned in the paper introduction, when a SMA wire temperature increases, it undergoes a phase transformation which leads to a change in both length and electrical resistance. This fact also implies that a constant power cannot be obtained by simply keeping the voltage or the current constant. Therefore, to drive the SMA fingers, a power control strategy is properly implemented in the experimental setup. For each bundle, reference values of power are calculated according to the wire producer recommendations (Fumagalli et al., 2009). Specifically, each SMA wire mounted in the finger structure (having 0.1 μm diameter) can be “safely” actuated by supplying 0.2 A to it. This means that, since all wires in the same bundle are electrically in parallel, each “sandwich bundle” needs 1.8 A to be actuated. This value represents an optimal trade-off between actuation speed and avoidance of microstructural damages due to prolongated SMA activation. The voltage, instead, is calculated according to each bundle length (Fumagalli et al., 2009). Therefore, the use of relatively short SMA bundles requires low input voltages and high input current values.

Since many nonlinear phenomena occur in the structure (e.g., contact friction between the SMA wires and the structure, influence of superelastic SMA), we decided to evaluate the force with a direct measurement of the load cell at the fingertip middle point.

The choice of the force application point is motivated by the fact that, during daily grasping operations, objects are mostly handled by using the hand fingertips. It is pointed out that the measured force is not constant along each finger phalanx, but it increases as the load cell is moved closer to the phalanx center of rotation. In order to measure an average representative value, the fingertip middle point is chosen for force evaluation, represented by the second circle starting from the top designed at the fingertip front part (see Figure 2a). The load cell is positioned in front of the fingertip, with a small gap between the two, in order to measure the prototype force as soon as the desired SMA bundles are activated. These experiments permit to measure the true finger force, and avoid at the same time the occurrence of undesired effects, e.g., the side-slipping due to bending of the soft structure, or additional contact forces like the friction between the finger prototype and the item to handle.

**Figure 7:**
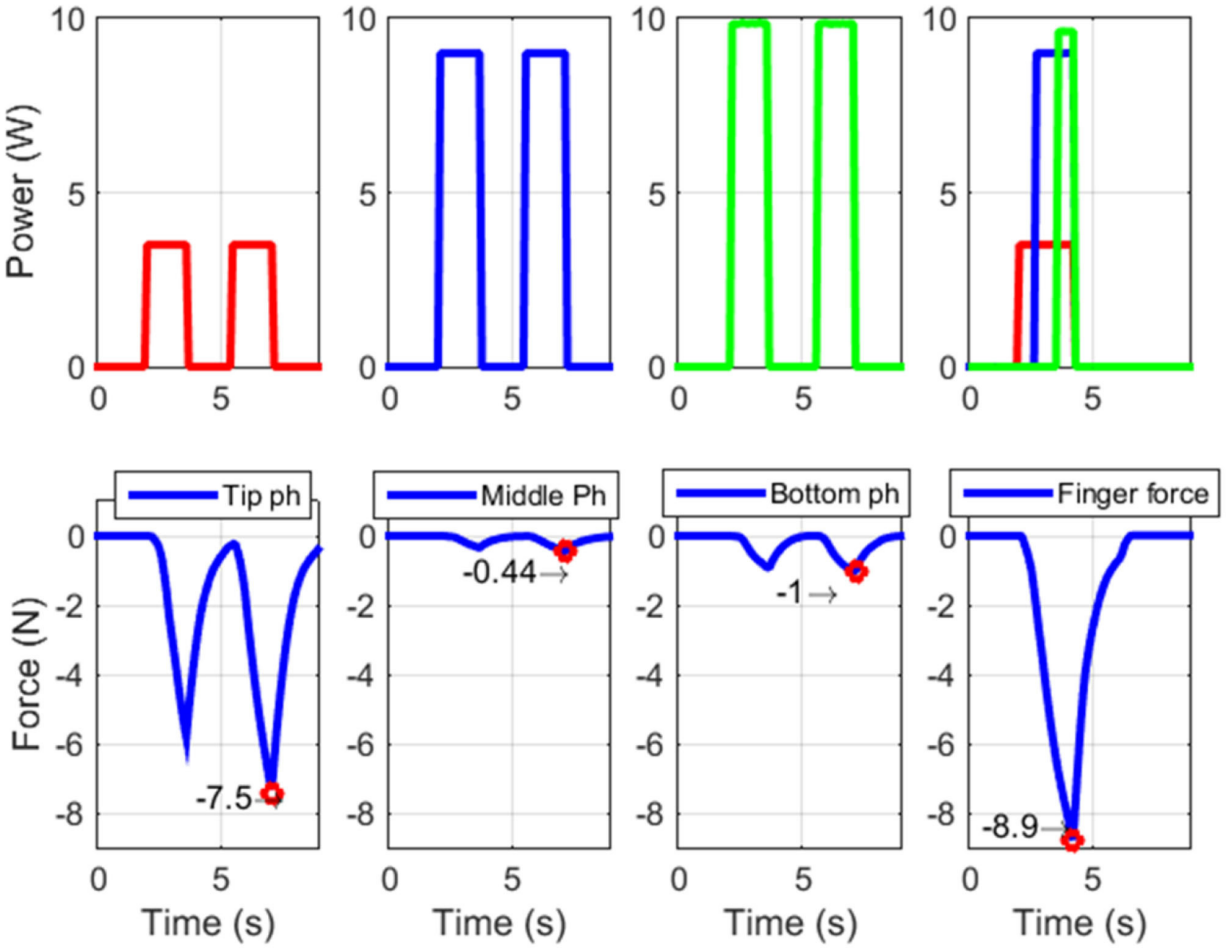
Forces evaluated at the fingertip actuating different protagonist SMA bundles at time, i.e., (left-hand side) tip phalanx, (center-left-hand side) middle phalanx, (center-right-hand side) bottom phalanx. (Right-hand side) Forces evaluated at the fingertip actuating all the three protagonist SMA bundles in the same time.

In addition, since the proposed test rig is entirely composed of commercially available components, it can be easily reproduced by other researchers, and used for the systematic characterization of different hand prototypes. The tests results are depicted in Figure 7. Moving from the left- to the right-hand side, the force of the protagonist bundle actuating the tip, middle, and bottom phalanx is shown, respectively. On the right-hand site of Figure 7, the finger overall force, obtained by activating all the protagonist bundles, is depicted. It is interesting to notice how the measured force of the top phalanx bundle is higher than the force of the bottom one, even if they have the same number of wires and the same SMA diameter. This phenomenon occurs because we are measuring the force far from the bottom phalanx center of rotation, and thus the force induced in the load cell has a large lever arm. On the other hand, note how the load cell prevents the motion of the SMA finger. As a result of the moment induced during the force measurement, the soft structure tends to deform according a different preferred configuration. This behavior effects the overall force component acting against the load cell. In the bottom row, left-hand side of Figure 7, the second force peak is slightly higher than the first. This is due to dynamic phenomena, such as residual heat occurring during the transient as well as due to the SMA wires hysteresis. The second peak represents the actual stabilized force value.

Figure 7 shows how the maximum achievable force for the SMA actuated finger, in the analyzed configuration, is almost 9 N. This value is comparable with the human finger one (Tubiana et al., 1998). From this force value, we can estimate a force of 45 N for the overall hand.

### Finger Motion

In this section, the maximum achievable displacement of each phalanx of the finger prototype is experimentally evaluated. The experimental bench is very similar to the one showed in Figure 6. It is based on a FPGA National Instruments cRIO-9074 real-time data acquisition interfaced with LabVIEW 2012. The FPGA is connected with a NI 9472 module.

**Figure 8:**
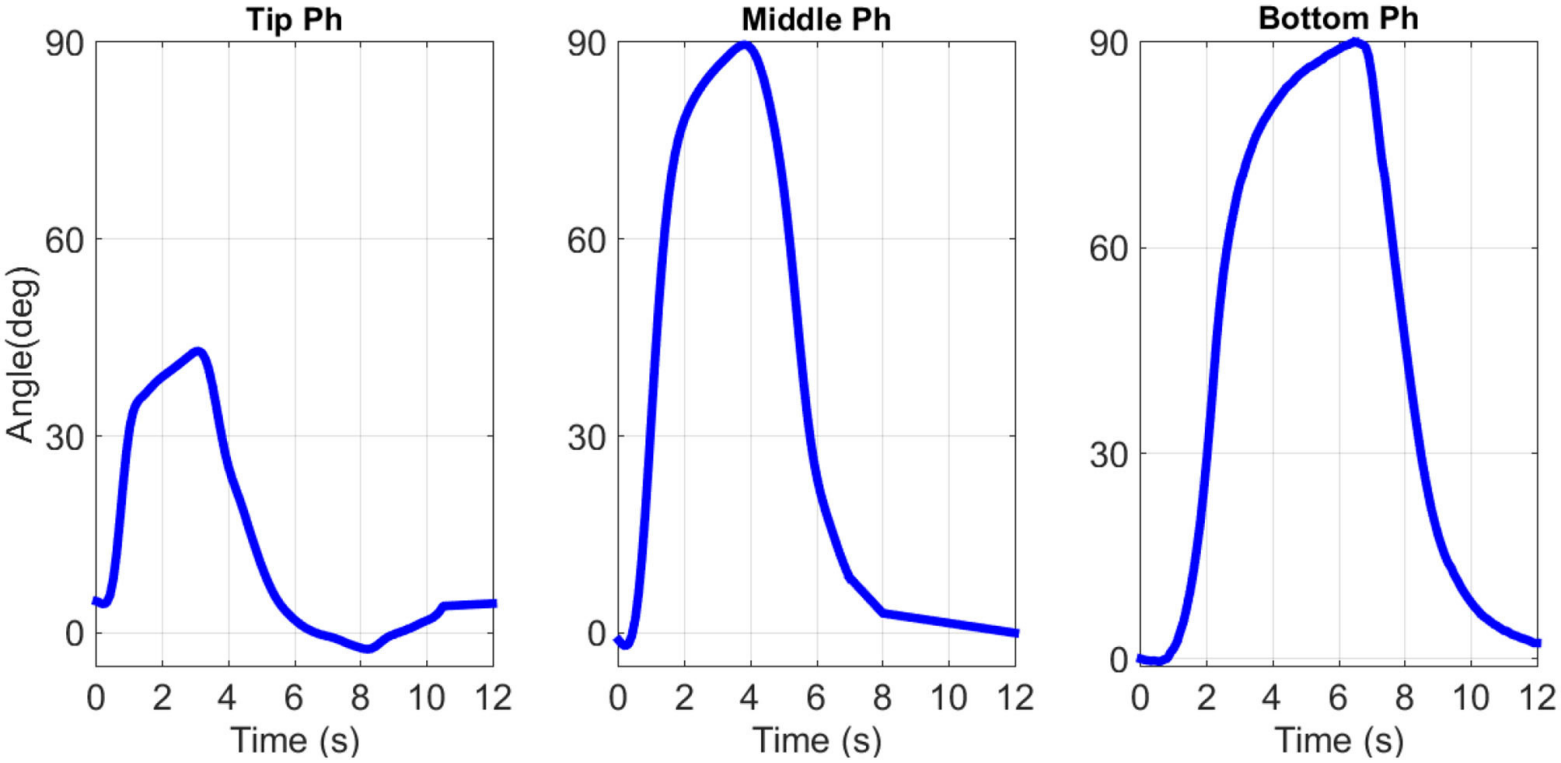
Finger phalanxes angular displacement. (Left-hand side) Top, (center part) middle, and (right-hand side) bottom phalanx displacement.

A custom electronic board is realized able to drive each bundle independently from the others. During each test, one channel of the NI 9472 is enabled at a time by the LabVIEW control logic, allowing to supply a desired power profile to the MOSFET connected to a corresponding bundle in the finger phalanx. In this way, each finger phalanx can be controlled independently of the other ones. In order to measure the finger motion, a Logitech webcamera is interfaced with LabVIEW and syncronized with the other NI module. At each sampling time, a video frame is recorded. The acquired video is then postprocessed with a MATLAB routine, and the signal describing the rotation angle over time is reconstructed. The antagonist SMA wires are activated at the beginning of each experiment, in such a way to ensure a fully straight initial configuration for each phalanx. Then, according to the desired input power signal designed in LabVIEW, the antagonist wires are deactivated while the protagonist SMA bundles are powered. The measured angles are displayed in Figure 8. From this picture, it can be verified that the prototype displacement performances are comparable with the human finger ones, and permits a wide range of motion (Tubiana et al., 1998). We can observe that each phalanx reaches its maximum rotation angle, without meeting any hard stop imposed by the structure (note how the angular displacement curve exhibit no plateaus). This is done by properly shaping (via trial and error) the amplitude of the input signals. We point out how the displacement of the human hand tip phalanxes varies from 45° up to 90°, moving from the ring to the little finger (Tubiana et al., 1998). On the other hand, during any grasping motion, the tip phalanx of each finger rarely reaches 90° of displacement. For these reasons, in all the commercial active prostheses, the tip finger phalanx joint is always designed to be fixed with an orientation of 20° with the respect of the middle phalanx (Belter et al., 2013). Since the tip phalanx favors grasping stability, in this work it has been designed to be movable.

As shown in Figure 5, reducing the length of the top bundles, and thus their actuation strain, makes it possible to increase the available space to integrate the bottom bundle. Therefore, 45° is chosen as top phalanx maximum rotation motion.

### High-Speed Activation

In the previous section, the maximum displacement of each finger phalanx is investigated. During each experiment, conservative values of current and voltage are used to power each SMA bundle (Fumagalli et al., 2009). In general, the SMA wire producers recomend “safe” current values to be supplied to the wires during long-term activation, in order to prevent any damage in the crystal structure.

**Figure 9:**
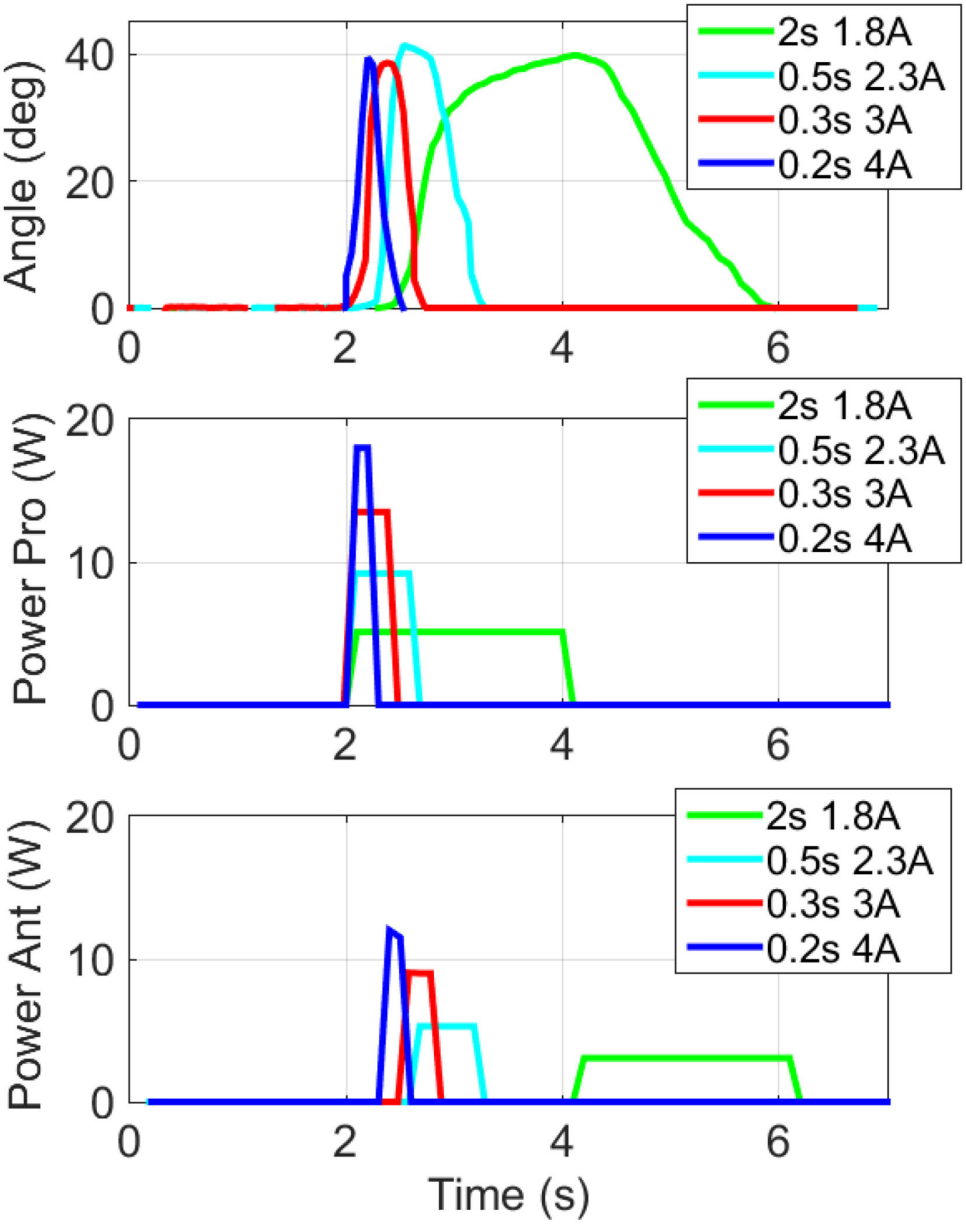
High speed activation of the protagonist and antagonist bundles in the fingertip. (Upper part) top phalanx angular displacement, (center part) input power protagonist bundle, (lower part) input power antagonist bundle. For each color line, the corresponding input current is depicted.

Besides this approach, it has been demonstrated that SMA wires can be activated much faster if powered via short pulses of high amplitude current (Vollach and Shilo, 2010; Vollach et al., 2016). This type of activation leads to no damage in the wire structure, if the pulses are shaped properly. The energy that has to be provided to the system with the short pulses needs to be sufficiently high to induce a fast phase transformation in the material. At the same time, the pulse duration should be long enough to let the wire heat up to its austenite finish temperature, which is around 90°C for Ni–Ti alloys. This method enables high activation speed of the SMA bundles using reasonable values of input energy. In order to reach activation speeds closer to the ones of human hand, the pulse activation method is tested on the SMA fingertip phalanx. For the experimental campaign, the test bench described in the previous section is employed, using several input current values having different amplitude and duration. At the beginning of each experiment, all finger bundles are not activated. The results of the experimental campaign are shown in Figure 9, where several measured angles and their correspondent input power signals are depicted. In this figure, the green line represents the phalanx angular displacement achieved using “safe” values of input power. In this case, the settling times for the rising and falling rotation steps ar Decreasing the duration and increasing the magnitude of the input power pulses leads to an increase of the prototype reactiveness. The blue line represents the fastest achieved motion, characterized by settling times for the rising and falling rotation steps of about 0.2 and 0.3 s, respectively. It is not possible to record more performant results with the available setup, due to frame rate limitations of the used web camera. Nevertheless, the achieved performance appears as comparable with the human hand during daily based operations (Tubiana et al., 1998). This approach can be used both in case of an impulsive action (e.g., for playing a piano) as well as for long time grasping tasks (e.g., for holding a bag).

In the first case, power profiles described by the blue line in Figure 8 (center and lower parts) can be used to supply the fingers bundles. In the other case, the pulse activation method must be followed by a constant power having a more conservative value (Figure 6, upper part), which need to be applied continuously to keep the finger at a fixed position. The value of such a constant power is defined by length, diameter, and number of wires welded in each bundle (Fumagalli et al., 2009) about 1.6 and 2 s, respectively.

This difference in time depends on the number of bundles involved in the motion, i.e., two protagonist and one antagonist SMA bundles. In particular, the value of the input current is related to the wire diameter (the bigger the diameter, the smaller the current required to heat the wire to a given temperature). Instead, the wire length influences the input voltage value (the longer the wire, the higher the voltage required to heat the wire to a given temperature).

In the finger prototype, the bundles are composed of several SMAs having identical diameter, therefore each wire receives the same amount of input current. On the other hand, each group of bundles (shown in different colors in Figure 5) has a different length, and therefore the input voltage amplitude differs in each bundle group (Fumagalli et al., 2009).

### SMA Hand Grasping Capabilities

As described in Section Design and Models, the finger-environment interface consists of rigid and elastic parts. The rigid part is represented by the 3D printed finger phalanxes front parts (Figure 2b). The elastic part is represented by the passive superelastic wires, which undergo large deformations when subjected to mechanical stresses.

**Figure 10:**
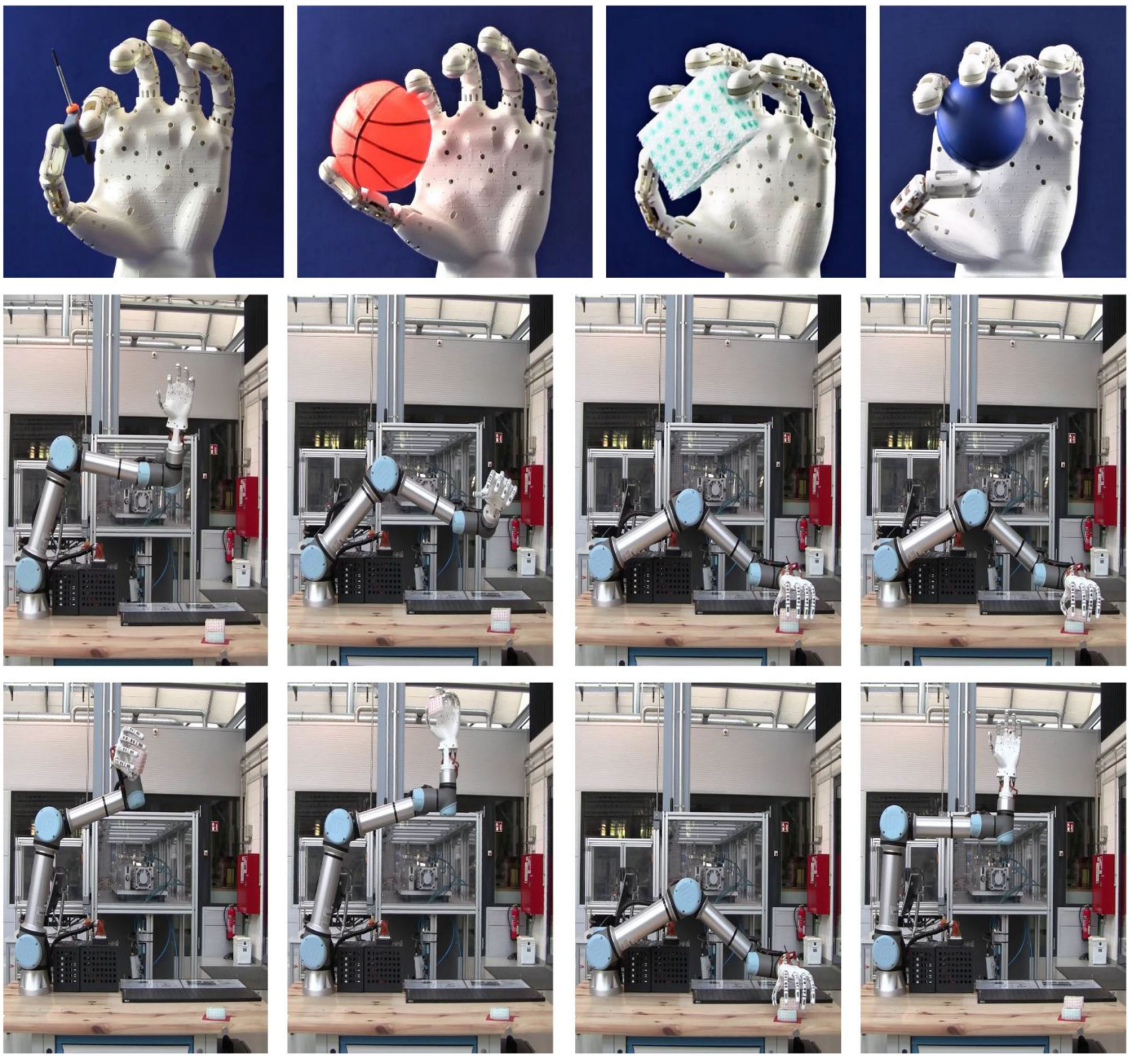
Complete hand grasping capabilities. In the pictures on the first row, starting from the left-hand side, the grasping of a screwdriver and of an orange ball using two fingers (lateral grasping) is depicted. In the same row, on the right-hand side, the grasping of a blue ball and of a rectangular small brick using four fingers is demonstrated. On the second and third rows, the grasping of a rectangular brick mounting the hand at the end-effector flange of a UR5^®^ collaborative robot arm is presented.

This design enables soft features in the hand structure, which are demonstrated in Video 1. In this video, the hand prototype is fixed to a breadboard laying on a table. In order to mimic a potential impact between the prototype and an obstacle, the prosthetic fingers are slightly hit by a human hand. In this case, the contact force results into an elastic force in the superelastic SMA. The finger joints deform accordingly, preventing any damage of both the prototype structure and the human hand. Once the obstacle (the human hand) is removed, the prototype fingers regain the original initial position. In Figure 10, first row, the grasping capabilities of the complete hand prototype are demonstrated. Four objects, having different size and shape, are chosen for the grasping experiments. In this way, several possible shapes (round/square, wide/thin) are investigated. During these experiments, the hand prototype is fixed to a commercially available breadboard using some screws and a custom basis. Each object is drawn near the prototype grasping area by the user, as shown in Video 1. In order to enable the independent motion of all the fingers, each phalanx is driven by a different output channel of an Arduino Mega^®^. Two different open-loop control strategies (one for the lateral and the other for the full hand grasping) are implemented in order drive each phalanx in a desired way. Prior to each experiment, the control strategy is manually tuned by the user. Starting from Figure 10, first row, left-hand side, the lateral grasping of the hand is depicted. Using only two fingers, the prototype is able to grasp both small and big objects, such as a screwdriver and a ball, respectively. This posture enables writing capabilities, e.g., if a pen is grasped. On the right-hand side of Figure 10, first row, a grasping process based on more fingers is demonstrated. In this way, it is possible to better handle objects with different shapes such as a small ball and a rectangular plastic brick. It is possible to notice, mostly from Figure 10, first row, right-hand side, how the thumb position during grasping feels unnatural. This is due to the fact that the current design lacks the DOF related to CMC joint.

In Video 1, in order to demonstrate the grasping stability, the prototype is disconnected from the breadboard and manually shaken by the user during the handling of the rectangular brick. All of the performed experiments demonstrate the handling capability of the presented hand prosthesis. It is remarked how an unstable grasping of extremely smooth objects is also observed. This is mostly due to a lack of friction force provided by the 3D printed material used for the fingers structure. In order to improve the prototype grasping ability, in future works a soft silicone finger cover will be developed to mimic the human skin and, in turn, improve the grasping stability.

To test the SMA actuated soft hand in a robotic scenario, the hand is mounted at the end-effector flange of a UR5^®^ collaborative robot arm. The robotic arm is programmed using the interface provided by the manufacturer, in order to reach the area where the object is positioned. At the same time, it is interfaced and synchronized with the microcontroller which drives the SMA hand.

In this way, once the robotic arm reaches the desired position, it enables the microcontroller to drive each hand finger according to a predetermined trajectory, and grasp the desired object. Video sequences of the performed experiments can be seen in Figure 10, second and third rows, and in Video 1. Once the desired object is grasped, the robotic arm is programmed to freely move in the air at different speed, demonstrating the effectiveness of the performed process.

### SMA Hand Performances Discussion

In the previous sections, the SMA Soft Hand performances have been described in terms of force, displacement, and reactiveness. In order to better clarify the advantages of the developed prototype, a performance benchmark with a number of recently developed myoelectric robotic hands is presented in this section. Pneumatic actuated prostheses are neglected from this comparative study, due to the structural disadvantage introduced by the technology (as discussed in the introduction section). In Table 2, all the chosen hand prototypes are listed and compared in terms of weight, size, DOFs, reactiveness, force, and structural complexity. We point out that, at present, no standard procedure is available to systematically characterize the force of this type of prototypes. For this reason, every manufacturer performs characterization experiments on the basis of custom-made sensors and test rigs. Since details on such measurement system are generally omitted, they are hard to reproduce in practice [5–7–14–18]. Nevertheless, since these measurements represents the only force values available from the literature, they will be used for the comparison realized in this section.

**Table 2:** Performance benchmark composed of recently developed myoelectric robotic hands and the SMA Soft Hand.

**Hand prosthesis name**	**Weight (g)**	**Overall size**	**DOF**	**Average finger speed (^**°**^/s)**	**Average force (N)**	**Finger couplings**	**Structure**
iLimb (2009)	600	180 mm long	6	~81.8	~68 (gripping)	Tendon linking MCP to PIP	Rigid
		80 mm wide					
		40 mm thick					
Bebionic (2011)	500	200 mm long	6	~96.4	~62 (gripping)	Linkage spanning MCP to PIP	Rigid
		90 mm wide					
		50 mm thick					
Michelangelo (2011)	420	Human hand size (no exact dimensions found)	6	~ 86.9	~70 (gripping)	Cam design with links to all fingers	Rigid
The smart Hand (2001)	530	Human hand size (no exact dimensions found)	16	~64	~40 (gripping)	Tendon/spring based	Rigid
Pisa/IIT Soft Hand (2015)	500	230 mm long	19	~90	~15 (gripping)	Synergies: 1 DC motor for all DOF	Soft
		235 mm wide					
		40 mm thick					
SMA Soft Hand (2020)	300	200 mm long	14	~450 (high-speed activation)	~45 (blocking)	No couplings	Soft
		116 mm wide					
		35 mm thick					

In this table, the iLimb, the Bebionic, and the Michelangelo hands are chosen among all the commercially available prostheses. Among all the myoelectric prototypes designed by research groups in recent years, the SmartHand (having stiff joints) and the Pisa/IIT Soft Hand (with soft joints) represent the most performing ones. In literature, the maximum weight of a human hand is quantified around 400 g (Belter et al., 2013). In Table 2, only the Michelangelo and the SMA Soft Hand do not overcome this value. In terms of dimensions, almost all of the prostheses are sufficiently similar to a real human hand.

The Smart hand represents the only exceptions, since it requires a 200 mm long arm to hold the strings and electric motors which drive the fingers. Therefore, its uses as prostheses for hand amputee presents some structural limitations. The DOFs of the commercially available hands are limited, since they combine the motion of the middle and of the bottom phalanx, keeping the tip phalanx fixed. The other prototypes, instead, offer up to 19 DOFs. The commercially available prostheses show similar performances in terms of responsiveness and gripping force, with typical values of about 85–90°/s and 65 N, respectively. These performances ensure good grasping and handling capabilities. The Smart Hand demonstrates sufficient fingers responsiveness (64°/s), while, despite exhibiting smaller forces than the commercial prostheses (40 N), its grasping capabilities are sufficient to perform daily life operations. Note also that all of the described prostheses have a stiff structure, which ensures robustness but also exposes the user also to possible involuntary damages. The use of soft prostheses helps the interaction between the user and the device, preventing potential injuries (Hirose and Umetani, 1979). For this reason, soft prostheses are preferable to stiff devices. Among the many prototypes described in literature, the Pisa/IIT soft hand denotes good grasping capabilities, a compact structure, and good finger responsiveness. As for many soft robots (Trivedi et al., 2008), the Pisa/IIT soft hand prototype lacks in grasping force (15 N), thus preventing the possibility to handle several useful objects in daily life. The SMA Soft Hand, instead, ensures dimensions and number of DOFs comparable to the other prototypes in Table 2. It is able to achieve a high finger responsiveness, by using the high-speed activation method. The obtained grasping force is three times larger the one of the Pisa/IIT Soft Hand.

We remark how the force value of the SMA Soft Hand has been evaluated via blocking force experiments, rather than via gripping tests designed *ad-hoc*. The grasping is a result of the shear force between the finger and the object which, in turn, depends on the normal force exerted by the finger through the SMA (i.e., the measured blocking force). The shear force used for gripping also depends on the friction between hand and object, which is further affected by many factors like the type of object and the cover material used for the hand. The reported normal force measurement represents, thus, an intrinsic figure of performance of the novel SMA soft structure. Additional tests aimed at characterizing the gripping force for different objects and cover materials (which, in turn, lead to different friction coefficients) would require a dedicated extensive investigation which lies beyond the scope of this paper.

Nevertheless, even if this force value is large enough to fulfill daily life operations, it is still far from the grasping force demonstrated by the commercial prostheses. Big advantages over the other hand prototypes are represented by its completely silent activation, its low weight (300 g), and its simple structure without transmission mechanisms which favors a lower price and a systematic assembling procedure.

## Conclusions

In this work, a new design for a bioinspired SMA actuated hand prototype with soft features has been presented. The described prototype can be used in biomedicine as a human prosthesis well-suited for human-robot collaboration tasks. The modular finger structure uses superelastic SMA wires in the joint in order to enable soft features. These wires have been arranged according to a X-shaped pattern, in order to avoid undesired joint configurations under induced lateral moments. Bundles of actuated SMA wires have been systematically manufactured using a welding method, which ensures an identical behavior for each wire in the same bundle. The use of SMA wires as actuators permits the design of a compact, lightweight, and silent hand device. A protagonist-antagonist configuration has been chosen for the SMA bundles, to enable a higher prototype reactiveness. In addition, the modular structure permits a simple and fast replacement of possible damaged SMA actuator bundles, without requiring the replacement of the entire finger assembly.

Several tests have been performed in order to demonstrate the prototype capabilities. Force measurements have been realized at the finger top phalanx, while activating all the protagonist bundles in the finger. A force of about 9 N per finger has been evaluated, which leads to an overall hand prototype force of about 45 N. Such a value is within the range of human hand force. The complete motion of each finger phalanx has also been recorded, showing also in this case values comparable with human fingers (45° for the tip phalanx, 90° for the middle and bottom phalanxes). Using a short pulse power excitation, a settling time between 0.2 and 0.3 s has been observed for both rising and falling phases of the finger rotation. The described method can be eventually used to enable better dynamic performances, provided that an adequate hardware is used. Objects with different sizes and shapes have been grasped by the SMA hand prototype, demonstrating its high gripping versatility. In relation to the state of the art, the presented prototype permits to achieve good performances in terms of force, reactiveness, and dexterity. At the same time, the use of SMA technology permits to manufacture low cost, lightweight, and silent prostheses. This fact allows to potentially increase the acceptance rate of the end user, especially in medical applications.

These performances are achieved at the expense of high input power, which is about 27 W per finger in case of simultaneous activation of all the phalanxes. This value is a consequence of the low energy efficiency of SMA wire actuators.

In future works, the thumb mechanism will be improved by adding also adduction/abduction and opposition movements in order to increase the hand dexterity. Additional experiments will then be performed to evaluate the handling capabilities when dealing with various types of daily life objects and hand payloads. Advanced dynamic models and simulation tools will also be developed. On the other hand, model-based control and self-sensing algorithms will be developed and implemented. In this way, we will be able to mimic the motion and gesture of a real human hand, to perform a more precise grasping of both fragile and small-size objects with a reduced sensory hardware, and eventually to exploit the material intrinsic hysteresis to further improve the energy efficiency (Riccardi et al., 2014).

## Data Availability Statement

The raw data supporting the conclusions of this article will be made available by the authors, without undue reservation.

## Author Contributions

FS: Design, manufacturing, and experimental results, GR, PM, and SS: Paper revision. All authors contributed to the article and approved the submitted version.

## Conflict of Interest

The authors declare that the research was conducted in the absence of any commercial or financial relationships that could be construed as a potential conflict of interest.
